# Editorial: Applications of 3D printing in cardiovascular medicine

**DOI:** 10.3389/fcvm.2023.1314071

**Published:** 2023-10-23

**Authors:** Vincenzo Positano, Enrico Ferrari, Shaojie Chen, Simona Celi

**Affiliations:** ^1^ BioCardioLab, UOC Bioingegneria, Fondazione Toscana G. Monasterio, Massa, Italy; ^2^ Division of Cardiac Surgery, Istituto Cardiocentro Ticino, Ospedale Regionale di Lugano, Lugano, Switzerland; ^3^ Cardioangiologisches Centrum Bethanien (CCB), Frankfurt Academy For Arrhythmias (FAFA), Kardiologie, Medizinische Klinik III, Agaplesion Markus Krankenhaus, Akademisches Lehrkrankenhaus der Goethe-Universität Frankfurt am Main, Frankfurt am Main, Germany

**Keywords:** 3D printing, cardiovascular diseases, surgical planning, catheter ablation, complex congenital vascular disease

**Editorial on the Research Topic** Applications of 3D printing in cardiovascular medicine

3D printing represents an emerging technology in the cardiology field, with emphasis on applications in cardiovascular surgery and interventional cardiology (1,2). The most established clinical use of 3D printing is pre-operative planning due to its ability to provide a tactile experience and improve patient communication (3). Future clinical uses include bioprinting heart patches, cardiac muscle (4), and devices such as heart valves (5) and catheters. The 3D-printing process includes several steps, such as medical image segmentation, creation of 3D meshes, choice of printing materials, and 3D printing technology. Given the complexity of the 3D printing process, all these aspects should be optimized to improve the clinical utility of 3D printing in the cardiovascular field. Hence, this field of study must encompass a wide range of research. This Research Topic focuses on recent developments and applications in the application of 3D printing to cardiology. In this editorial article, we summarize the featured work in this issue.

The studies collected in this Research Topic are aimed to address different key issues in the 3D printing process, including three technical papers and three case studies. The first technical paper focuses on the characterization of 3D printing materials, which represents a fundamental step toward the use of 3D printing in the production of biocompatible devices. Moving to 3D printing of medical devices to direct patients’ treatment, appropriate tests should be conducted to ensure both the safety and effectiveness of developed devices. In this view, Cappello et al. investigate the thermal properties of two biocompatible materials (MED625FLX and TPU95A) suitable for 3D printing technology. The two biomaterials are candidates for the development of a cardiac surgical guide for radiofrequency ablation (RFA) treatment. Both biomaterials tested were demonstrated inappropriate for unipolar ablation because of temperature concerns, while MED625FLX could be used for bipolar ablation. The paper from Aigner et al. is an interesting example of the development of 3D *in vitro* patient-specific phantoms to be inserted in a mock circulatory loop (MCL) (6) for blood flow simulation. MCLs are *in vitro* experimental systems that provide continuous, realistic pulsatile flows to simulate different physiological or pathological conditions. They are useful for testing cardiovascular assist devices or to simulate interventional procedures if the blood flow characterizations is required. MCLs allow precise and reproducible measurement of flow parameters, by sensors or image modalities, such as ultrasound, particle image velocimetry (PIV), and 2D phase contrast of 4D-Flow MRI. The authors simulated aortic flow in a patient with severe aortic regurgitation after transcatheter aortic valve implantation (TAVI), to investigate the presence of paravalvular leaks (PVL). As the developed mode was MR-compatible, the flow was assessed by high-resolution 4D-flow MRI. The study demonstrated the ability of 4D-flow MRI in the identification of PVL. Depending on the kind of measurement to be performed, the properties of the 3D model to be inserted in the MCLs should be accurately defined. For instance, the PIV technique requires the use of a transparent material because particles should be visible to the optical acquisition device. If magnetic resonance is used, the model should be magneto-compatible to avoid image artifacts. In the paper from Antonuccio et al., the authors developed a 3D model that can be used with both PIV and MR image modalities. Casting techniques were employed to develop ascending aorta aneurysm models suitable for *in vitro* hemodynamic investigations, such as PIV measurements or medical imaging. The casting technique allows the development of 3D models with silicone-based materials, which are the most suitable for several applications but are challenging to be directly printed (7). The authors demonstrated the compatibility of the developed model with several image modalities (MRI, CT, Doppler US) and PIV.

The three case studies included in the Research Report describe different approaches involving 3D printing support in cardiac surgery planning. These papers confirm the growing interest in the use of 3D printing to refine anatomic diagnosis before surgery (8), especially in congenital diseases. Giugno et al. describe the use of an *in vitro* planning platform to evaluate the feasibility of a stenting procedure under fluoroscopic guidance in a case of pediatric mid-aortic syndrome. A translucent flexible 3D-printed patient-specific model was obtained from CT images and the implant of two stents was simulated. The percutaneous procedure was successfully performed, replicating the strategy tested *in vitro*. In the paper of Zhang et al., a personalized 3D-printed model was used for preoperative assessment in a complex pediatric case involving congenitally corrected transposition of the great arteries (ccTGAs). With the guidance of 3D printing, the operation was successful, with a good recovery of the patient. Finally, a rare case of cardiac metastatic uterine intravenous leiomyomatosis was described by Chen et al. A patient-specific pre-operative 3D-printed model obtained from CT images was used to plan the surgery. 3D-printing was useful in intuitive and accurate understanding of the tumor size and in defining the relationship between the tumor and the adjacent tissues.

In all three cases, pre-operative evaluation and guidance of 3D printing demonstrate their usefulness in surgery in patients with rare anatomical structures and high surgical risk.
